# Special Issue “Role of NRF2 Pathway in Chronic Diseases”

**DOI:** 10.3390/ijms27104460

**Published:** 2026-05-16

**Authors:** Ángel Juan García-Yagüe

**Affiliations:** 1 Department of Biochemistry, School of Medicine, Autonomous University of Madrid (UAM), 28029 Madrid, Spain; ajgarcia@iib.uam.es; Tel.: +34-91585-4382-4383; 2 Instituto de Investigaciones Biomédicas “Sols-Morreale” (CSIC-UAM), C/Arturo Duperier, 4, 28029 Madrid, Spain; 3 Instituto de Investigación Sanitaria La Paz (IdiPaz), 28046, Madrid, Spain; 4 Centro de Investigación Biomédica en Red de Enfermedades Neurodegenerativas (CIBERNED), 28031 Madrid, Spain

The progressive increase in life expectancy over the past century has been accompanied by a parallel rise in the prevalence of chronic diseases, including metabolic, cardiovascular, neurodegenerative, and inflammatory disorders. Despite their apparent heterogeneity, these conditions share common pathogenic mechanisms, notably oxidative stress, chronic low-grade inflammation, and metabolic imbalance. In this context, the transcription factor NRF2 has been identified as a central regulator of cellular homeostasis and a promising therapeutic target within a systems medicine framework [1]. Traditionally, chronic diseases have been approached from an organ- or symptom-based perspective. However, recent advances in network medicine have enabled a paradigm shift toward mechanism-based classification, integrating molecular interactions and shared biological pathways [2,3]. NRF2 exemplifies this transition, as it occupies a key node within a network of cytoprotective responses that are broadly dysregulated across multiple chronic pathologies.

At the molecular level, NRF2 functions as a master transcriptional regulator of cellular defense mechanisms. It controls the expression of over 250 genes containing AREs, which encode proteins involved in redox balance, detoxification, metabolism, and proteostasis [4,5]. These include enzymes responsible for glutathione synthesis, NADPH regeneration, and xenobiotic metabolism, as well as components of the proteasome and autophagy machinery [1]. Through this extensive transcriptional network, NRF2 orchestrates a coordinated response to oxidative and electrophilic stress, thereby maintaining cellular integrity.

Beyond its molecular regulation, the integration of NRF2 within a network-based analysis reveals that NRF2 is functionally connected to numerous proteins involved in metabolic pathways, inflammatory responses, and gene regulation, providing critical insights into its role in disease [2,3]. Importantly, perturbations within this network are associated with a wide spectrum of diseases, including cancer, diabetes, kidney diseases, cardiovascular disorders, respiratory diseases, and neurodegeneration [1]. This interconnectedness underscores the concept that diverse clinical phenotypes may arise from shared molecular dysfunctions centered around NRF2 signaling.

A critical aspect of NRF2 biology is its role in the resolution of inflammation, a hallmark of chronic disease. Persistent inflammatory responses are often driven by excessive production of ROS and RNS, which amplify signaling pathways such as NF-κB [6]. NRF2 counteracts these processes by restoring redox balance and modulating inflammatory signaling. Furthermore, NRF2 influences immune cell function and phenotype. It promotes an anti-inflammatory profile, enhances antioxidant capacity through glutathione metabolism, and modulates T-cell responses [1,7]. Collectively, these effects help to limit tissue damage and facilitate the resolution of inflammation.

In the central nervous system, NRF2 has been identified as a critical factor in neuroprotection. Neurodegenerative diseases such as Alzheimer’s disease [8] (Contribution 2), Parkinson’s disease [9], and amyotrophic lateral sclerosis [10] are characterized by oxidative stress, neuroinflammation, and impaired proteostasis, processes tightly regulated by NRF2. Experimental and clinical evidence suggests that NRF2 activation attenuates neuronal damage by enhancing antioxidant defenses, modulating astroglial and microglial activation, and promoting clearance of toxic protein aggregates.

Importantly, NRF2 also contributes to the maintenance of BBB integrity [11]. The BBB is highly sensitive to oxidative stress and inflammation, which can disrupt tight junctions and increase permeability. NRF2 activation in endothelial cells enhances the expression of antioxidant and cytoprotective genes, preserving barrier function and limiting neuroinflammatory infiltration [11]. Conversely, impaired NRF2 signaling has been associated with BBB dysfunction, exacerbating neurodegenerative processes and altering the angiogenesis pathway through TIE2 receptor (Contribution 1). Thus, NRF2 represents a key regulator not only of neuronal survival but also of neurovascular unit integrity.

In the kidney, NRF2 plays a protective role against CKD, a condition characterized by progressive loss of renal function driven by oxidative stress, inflammation, and fibrosis (Contributions 4 and 5). Experimental models have shown that NRF2 deficiency exacerbates renal injury, while its activation reduces oxidative damage, inflammation, and fibrotic remodeling (Contribution 4). In diabetic nephropathy and other forms of CKD, NRF2 modulates the expression of antioxidant enzymes and suppresses pro-fibrotic signaling pathways, suggesting its therapeutic potential in slowing disease progression (Contribution 4).

Similarly, NRF2 is critically involved in liver physiology and pathology. The liver is continuously exposed to xenobiotics and metabolic stress, making NRF2-mediated detoxification pathways essential for maintaining hepatic homeostasis. NRF2 protects against drug-induced liver injury, steatosis, and progression to NASH by regulating antioxidant defenses and lipid metabolism [12]. However, persistent oxidative stress in chronic liver disease often leads to an insufficient or maladaptive NRF2 response, highlighting the need for therapeutic modulation [13].

Beyond these systems, NRF2 also plays significant roles in cardiovascular and respiratory diseases. In the vasculature, NRF2 regulates endothelial function and protects against atherosclerosis by reducing oxidative stress and inflammation. In the lung, NRF2 deficiency increases susceptibility to chronic obstructive pulmonary disease and pulmonary fibrosis, whereas its activation restores antioxidant capacity and improves tissue repair mechanisms [1].

The relevance of NRF2 in chronic diseases is also supported by genetic and clinical evidence. Polymorphisms in the *NFE2L2* gene have been associated with altered susceptibility to inflammatory and metabolic disorders, indicating that even subtle changes in NRF2 activity can have significant pathological consequences [14]. Moreover, dysregulation of NRF2 signaling has been observed in patient samples across multiple disease contexts, often reflecting either insufficient activation or compensatory upregulation in response to chronic stress [1].

Taken together, these findings position NRF2 as a central hub linking oxidative stress, inflammation, and metabolic dysfunction across a broad spectrum of chronic diseases. This integrative view aligns with the principles of systems medicine and supports the development of mechanism-based therapeutic strategies targeting NRF2 and its regulatory network.

This Special Issue has gathered a diverse collection of original research articles and reviews that collectively advance our understanding of NRF2 biology in chronic diseases. The included contributions highlight novel regulatory mechanisms of NRF2 activity; explore its role in specific pathological contexts, including neurodegeneration or chronic kidney disease; and discuss alternative traditional health practices with similar preventive effects to exercise (Contribution 3). Thus, these works underscore both the complexity and the translational potential of NRF2 signaling. We hope this Special Issue provides a comprehensive, up-to-date perspective that will stimulate further research and foster the development of innovative therapeutic approaches targeting NRF2 in chronic diseases.

In conclusion, the NRF2 pathway represents a unifying mechanism underlying diverse chronic pathologies. Its role as a master regulator of cellular defense, combined with its integration into complex molecular networks, underscores its potential as a cornerstone for future therapeutic strategies. Continued research into NRF2 biology will be essential for translating these insights into clinical practice.

## Abbreviations

ARE	Antioxidant response elements
BBB	Blood–brain barrier
CKD	Chronic kidney disease
NADPH	Nicotinamide adenine dinucleotide phosphate
NASH	Non-alcoholic steatohepatitis
NF-κB	Nuclear factor kappa-light-chain-enhancer of activated B cells
NRF2	Nuclear factor erythroid 2–Related Factor 2
ROS	Reactive oxygen species
RNS	Reactive nitrogen species
